# The role of flour type and feeding schedule on the sourdough microbiome

**DOI:** 10.1128/spectrum.02380-25

**Published:** 2025-11-25

**Authors:** Sima Taheri, Enrique Schwarzkopf, Hanna L. Berman, Nathan Brandt, Jessica McNeill, Nico Sevier, Margot Ruffieux, Robert R. Dunn, Caiti Smukowski Heil

**Affiliations:** 1 Department of Food Science, Gorgan University of Agricultural Sciences and Natural Resources 123290, Gorgan, Iran; 2 Department of Applied Ecology, North Carolina State University 6798 , Raleigh, North Carolina, USA; 3 Department of Biological Sciences, North Carolina State University 6798 , Raleigh, North Carolina, USA; University of Minnesota Twin Cities, St. Paul, Minnesota, USA

**Keywords:** sourdough, flour, 16S, ITS, microbial ecology, *Kazachstania*, lactic acid bacteria

## Abstract

Sourdough starters are fermentations of various grains by bacteria and yeast and are of worldwide economic and cultural importance. Sourdoughs are sometimes spontaneously inoculated, and their resident microbial communities are in part shaped by environmental factors, potentially including flour, water, air, human microbiota, equipment, geography, and temperature. The number of different genera of bacteria and yeast found in sourdoughs is large; however, only a handful of species typically dominate an individual sourdough starter. Understanding how and why certain species form a mature climax community in a particular environment is a key question in microbial ecology. To investigate this question, we used a meta-barcoding approach and tested whether different baking flours (all-purpose, bread, and whole wheat) and frequency of feeding, also known as backslopping, shape the sourdough starter microbial community over the course of one month. We found that the yeast genus *Kazachstania* rapidly rose in frequency and became the most abundant yeast in all starters, regardless of flour type or feeding schedule. In contrast, flour type did affect the bacterial community. Mature sourdoughs all contained the bacterial genera *Companilactobacillus*, *Levilactobacillus*, *Lactiplantibacillus*, *Furfurilactobacillus*, and *Acetobacter*, with *Companilactobacillus* detected at higher relative abundance in whole wheat flour and *Levilactobacillus* detected at higher relative abundance in bread flour. We conclude that flour can shape the microbial community of sourdough and has potential implications for functional traits.

**IMPORTANCE:**

How organisms disperse and colonize new environments is central to our understanding of biodiversity. Sourdough, the often spontaneously inoculated fermentation of grains by bacteria and yeast, represents a great system to test and observe how microorganisms come to inhabit a particular niche. In our study, we investigate how environmental parameters such as flour type and feeding frequency influence the microbial community. We find that the common sourdough yeast genus *Kazachstania* is most abundant in all starters regardless of treatment, but we also find a significant effect of flour type on the lactic acid bacteria composition of the sourdough starters. This work shows how the environment can impact the presence and abundance of particular microorganisms and prompts future studies to test how particular lactic acid bacteria species can specialize on certain resources.

## INTRODUCTION

Sourdough describes the fermentation of grains and water by a mixture of yeasts and bacteria for the production of bread and related products. This type of fermentation is an ancient practice, with the earliest evidence of yeast and bacteria in sourdough from China and Egypt in the first and second millennia BC (1, 2), though earlier archaeobotanical remains suggest a more ancient origin for breadmaking and the processing of vegetal materials for consumption (3–7). Sourdoughs are maintained through “backslopping,” which entails discarding a portion of the sourdough starter and providing new grains and water (8–10). There are many variations on this process across culture, geography, time, and scale of production (11–14). Various forms of sourdough are produced by cultures across the globe, and leavened bread serves as a staple food source for billions of people, making it a central economic and cultural commodity.

The microbial community of sourdough contributes to a number of important traits including leavening, aroma, crumb structure, nutrient availability, and shelf stability (8, 15, 16). Culture-dependent isolation and DNA sequencing-based approaches have identified over 60 genera of bacteria and over 80 species of yeast from sourdoughs around the world (8, 17, 18). While a large number of species may be found across sourdough samples (what ecologists would term “gamma diversity”), the local/alpha diversity of each individual sourdough is typically low and dominated by a few species of bacteria and one to two species of yeast (11). Bacterial species are typically dominated by lactic acid bacteria (LAB) in the family Lactobacillaceae, with *Lactiplantibacillus plantarum*, *Levilactobacillus brevis*, *Fructilactobacillus sanfranciscensis*, and *Limosilactobacillus fermentum* most commonly identified (19), and *Saccharomyces cerevisiae*, followed by *Kazachstania humilis* and other *Kazachstania* species, the most prevalent yeasts (18, 20, 21).

Sourdough starters have become a model system—or, as some ecologists might put it, microcosms—for studying succession—the way species and habitats change over time—and for understanding abiotic and biotic interactions (9, 22). The community of sourdoughs is initially influenced by dispersal and further shaped by competition and selection associated with the continuous backslopping process. Raw materials (i.e., flour and water), bakers’ hands, and the surrounding air and surfaces have all been identified as sources for the sourdough microbial community, which—combined with bakers’ practices and other environmental factors such as temperature—shapes the sourdough microbiome (11–13, 23–31).

The substrate has been a focus of sourdough studies, both as a likely source of microbes and as an important factor in nutrient availability for the community. Diverse substrates are utilized in sourdoughs, including wheat, rye, barley, teff, millet, sorghum, buckwheat, amaranth, rice, and legumes. However, the microbial communities present on grains prior to milling and forming a sourdough often differ from those of mature sourdough starters, at least in terms of detectable microorganisms (31–34). For example, *S. cerevisiae* is rarely detected in flours, even though it is highly abundant in sourdough starters worldwide, either because it is absent from flours or because it is present at levels below the detection limits of culture-based or sequencing-based methods. Recent population genomic work has illustrated that certain *S. cerevisiae* strains are uniquely found in (typically mature) sourdough, and that these strains differ from strains found in commercial baking, other fermentations, and wild environments (20, 35, 36). The continuous competition and associated selection that arise from backslopping have led to adaptation to the sourdough environment for certain strains of *S. cerevisiae*, and this is also reflected in particular strains of lactic acid bacteria, though whether there is adaptation to particular flour substrates remains unknown. The effects of flour type on microbial community composition have been mixed, with some studies finding the same genera/species regardless of flour type (37–39), while others report a significant effect of flour on the mature community composition (10, 24, 31, 40–43). A large-scale study of over 500 sourdough starters (mostly from North America) found only modest effects of geography and flour type on species prediction (11).

In this study, we investigate the role of flour type and feeding schedule on the microbial communities of sourdough starters in a lab environment. We used all-purpose, whole wheat, and bread flour, all of which are commonly used for sourdough breadmaking in North America. Starters were fed either daily or every other day. Using meta-barcoding, we show that flour and feeding schedule had little effect on the fungal community; regardless of treatment, the climax community was primarily composed of species in the yeast genus *Kazachstania*. In contrast, the relative composition of the bacterial community varied by flour type, with all starter communities consisting of *Acetobacter*, *Companilactobacillus*, *Lactiplantibacillus*, *Furfurilactobacillus*, and *Levilactobacillus* at different proportions depending on substrate.

## RESULTS

### Raw whole wheat flour differs in fungal community compared to all-purpose and bread flours

**Fig 1:**
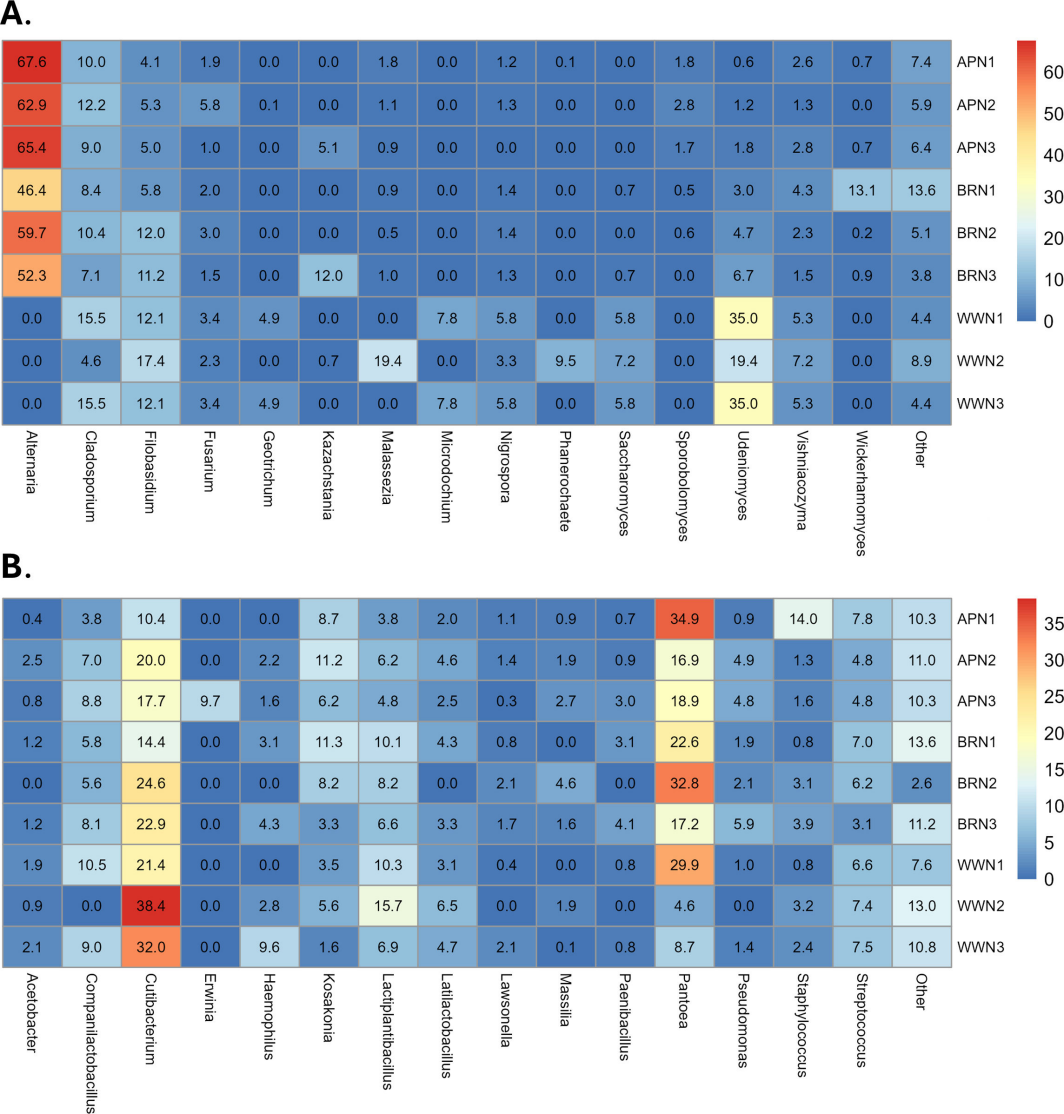
Fungal and bacterial composition of each replicate of different raw flour types at the taxonomic level of genus: all-purpose flour (APN), bread flour (BRN), and whole wheat flour (WWN) in three replicates (1–3). (**A**) Heat map comparing the average relative abundance (%) of fungal taxa detected in each type of flour. (**B**) Heat map comparing the average relative abundance (%) of bacterial taxa detected in each type of flour.

We first investigated the composition of microbial communities of the three types of commercial flour (all-purpose, bread, and whole wheat flour) prior to the formation of sourdough by amplicon sequencing of the 16S V3V4 (bacteria) and ITS (fungi) loci (Fig. 1). The flour types selected represent the most commonly used and accessible flours in the United States and include a range of protein contents (all-purpose flour: ~10.5%; whole wheat flour: ~13.8%; bread flour: ~12.3%). Based on relative read abundance of the raw flour, *Alternaria*, a taxon containing common wheat/cereal pathogens, was the most abundant fungal genus in all-purpose (~65%) and bread flour (~52%), but was not detected in whole wheat flour (0%) (Fig. 1A). Instead, *Udeniomyces* was present in whole wheat flour (~35%). *Cladosporium* and *Filobasidium*, common mold taxa that include both endophytes and plant pathogens, were the next most abundant genera in all three types of flour. Known sourdough yeast genera*—Saccharomyces*, *Kazachstania*, and *Wickerhamomyces*—were identified at low relative abundance (proportional read number) when present. *Saccharomyces* was present in all three whole wheat samples and in two of the bread wheat samples, while *Kazachstania* was present in one sample from each flour type. Notably, both *Saccharomyces* and *Kazachstania* were not detected from a number of flour samples in which they later appeared once those flour types were fermented. Within-sample (alpha) diversity of genera did not differ among flour types (Fig. S1). Whole wheat flour fungal composition was significantly different from all-purpose and bread flour (PERMANOVA *P* < 0.05; Fig. S2), but other comparisons were not significant.

The composition of bacteria did not differ within or among raw flour types (Fig. S1 and S2). Regardless of flour type, bacterial communities in flour were characterized by two taxa, the commonly plant-associated *Pantoea* (44) and the human skin-associated *Cutibacterium* (Fig. 1B). The presence of *Cutibacterium* might indicate contamination introduced through the sampling process. Alternatively, their presence could also be associated with grain production, milling, and transportation and hence reflect the normal ecology of flour. *Companilactobacillus* and *Lactiplantibacillus*, two genera of lactic acid bacteria (LAB) that typically are found in mature sourdoughs, were identified across raw flour types at low relative abundance. *Furfurilactobacillus* and *Levilactobacillus* were not among the most common bacterial taxa present in raw flour, although they did appear in higher abundance after fermentation. *Acetobacter*, a genus of acetic acid bacteria (AAB) often present in mature sourdoughs, though sometimes in association with off flavors, was present in all but one raw flour sample at a relatively low abundance (≤2.5%). Overall, the fungal and bacterial genera we found in flour largely overlap with those identified in previous studies of flours used in sourdough starters (31, 34, 41, 45–47).

### Flour type affects bacterial but not fungal communities in mature sourdough starters

To investigate how flour type and feeding schedule impact microbial communities in sourdough, we set up individual starters with all-purpose (AP), bread (BR), or whole wheat (WW) flour. Each flour type had three replicates fed daily (D) and three replicates fed every other day (E) for a total of 18 starters. We maintained these starters for 28 days, sampling DNA at five time points to monitor succession. Alpha diversity (observed amplicon sequence variant [ASV] richness and Shannon index) generally decreased from day 0 samples to day 28 samples (Fig. S6), with day 28 fungal and bacterial communities having similar measures of alpha diversity across flour types and feeding schedules (Fig. S4; Kruskal–Wallis with Bonferroni correction, *P* > 0.05, for all comparisons, except observed richness daily samples, *P* < 0.05, for yeast between all-purpose and bread flour types).

**Fig 2:**
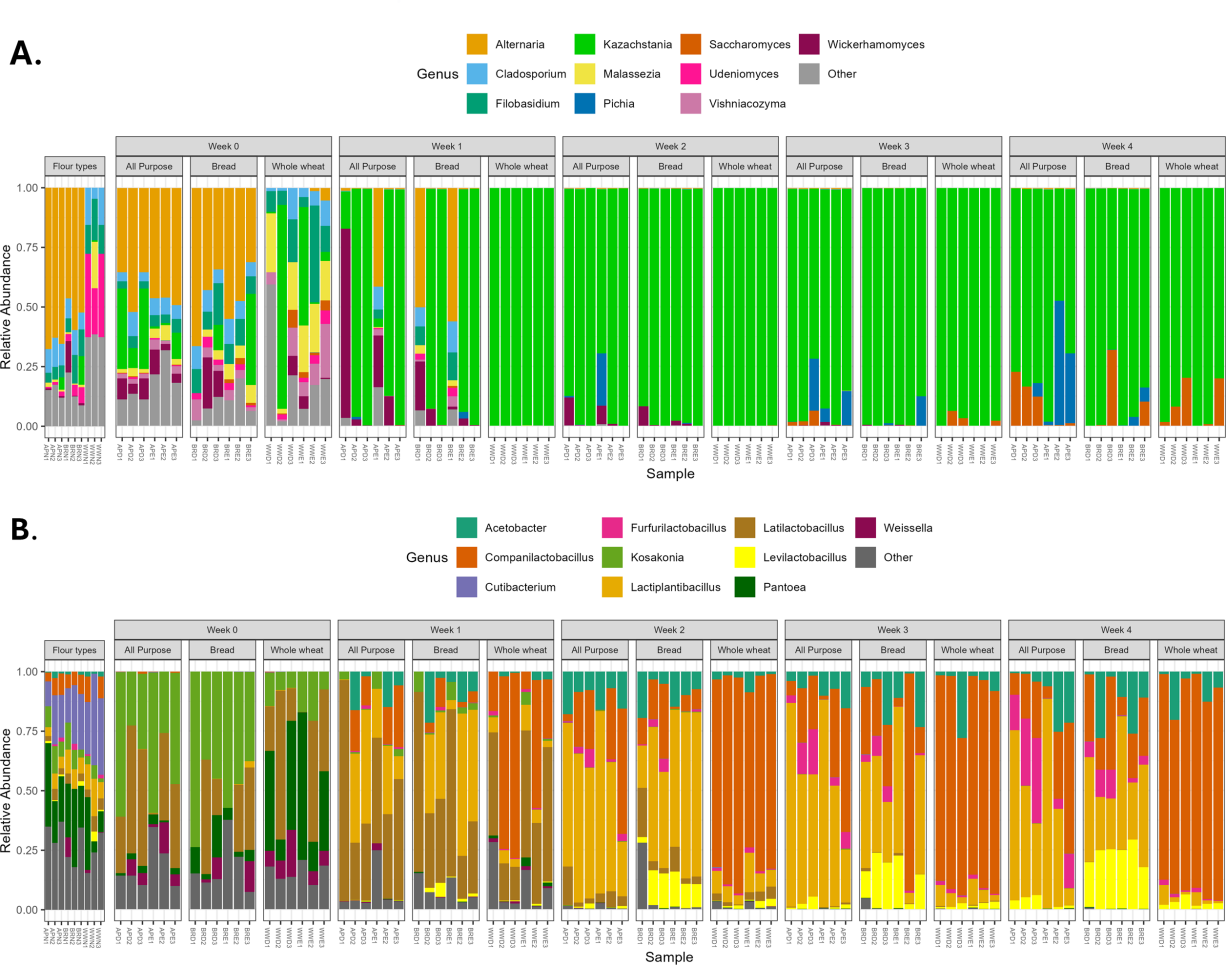
Relative abundance (%) of the ten most abundant fungal genera (**A**) and bacterial genera (**B**) for different sourdoughs with three types of flour—all-purpose (AP), bread (BR), and whole wheat (WW)—and maintained under two feeding schedules: daily (D) and every other day (E), shown through time. Less abundant genera are collapsed into “other” in gray. The full list of taxa identified can be found in Tables S3 and S4.

**Fig 3:**
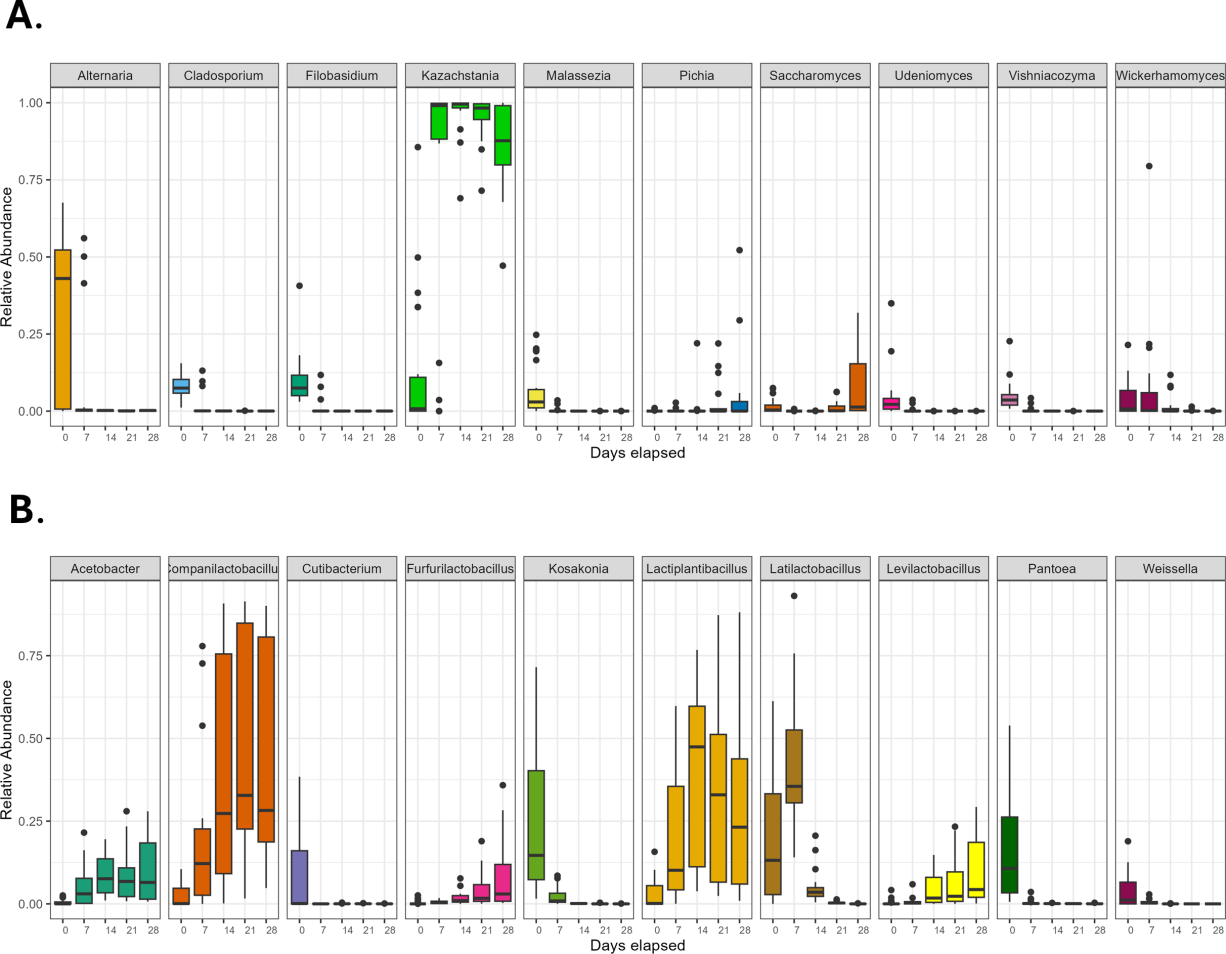
Relative abundance of top 10 fungal (**A**) and bacterial (**B**) genera combined for all samples through time. (**A**) We detected significant differences in the relative abundance of six of the top ten fungal genera over the course of one month, including decreases in *Alternaria*, *Cladosporium*, *Filobasidium*, and *Malassezia* and increases in *Kazachstania* and *Saccharomyces* (Kruskal–Wallis with Bonferroni correction, *P* < 0.005). (**B**) We detected significant differences in the relative abundance of all ten of the top bacterial genera over one month, including decreases in *Cutibacterium*, *Kosakonia*, *Pantoea*, and *Weissella* and increases in *Acetobacter*, *Companilactobacillus*, *Furfurilactobacillus*, *Lactiplantibacillus*, *and Levilactobacillus* (Kruskal–Wallis with Bonferroni correction, *P* < 0.0005). *Latilactobacillus* increased during the first week and then decreased in abundance over the remaining three weeks (Kruskal–Wallis with Bonferroni correction, *P* < 1.74e-13).

All starters underwent a rapid transition in community composition in the first 7–14 days. Most notably for fungal species, *Alternaria* rapidly and dramatically decreased after one week of feeding, while the relative abundance of *Kazachstania* increased; it rose quickly in all samples regardless of flour type and feeding schedule (Table S5; Fig. 2 and 3; Fig. S3). This was true even in the starters in which *Kazachstania* was not detected on day one. However, by day 28, *Saccharomyces* increased in frequency, including in samples and flours where it was not initially detected. We cannot exclude the interesting possibility that *Saccharomyces* might have become more prevalent if passaging continued beyond 28 days. Feeding frequency and flour type had little effect on fungal composition; all replicates converged quite rapidly, with *Kazachstania* emerging as the predominant yeast genus detected (Fig. S5).

In contrast to the results for fungi, flour type did alter the mature sourdough bacterial community (Fig. S5, *P* = 0.0.001 by PERMANOVA). *Pantoea*, *Weissella*, *Kosakonia*, and *Cutibacterium* all decreased during the first seven days of sourdough passaging (Table S5; Fig. 2 and 3). Similar to other sourdough studies, the succession of the community was marked by increases in acetic acid bacteria of the genus *Acetobacter*, as well as lactic acid bacteria of the genera *Companilactobacillus*, *Furfurilactobacillus*, *Lactiplantibacillus,* and *Levilactobacillus* (Table S5; Fig. 2 and 3). By day 28, all starters were primarily composed of these five genera, although their proportions depended on flour type (Fig. 2B). Whole wheat sourdough starters were distinguished by the comparatively high presence of *Companilactobacillus*. All-purpose and bread sourdough had more similar profiles to one another, with relatively more *Levilactobacillus* in bread flour and relatively more *Furfurilactobacillus* and *Lactiplantibacillus* in all-purpose flour (Fig. 2B). The relative abundance of *Acetobacter* was similar across flour types. We were unable to identify species-level designations with this 16S data set; however, the ASVs that we observed match sequences found in characteristic sourdough bacteria species, including *Companilactobacillus paralimentarius, Lactiplantibacillus plantarum*, and *Levilactobacillus brevis*.

## DISCUSSION

In this study, we sought to identify how environmental variables, such as flour type and feeding schedule, impact the fungal and bacterial community of sourdough starters. Feeding schedule is a variable frequently discussed and varied by bakers. Our experimental design employed two feeding schedules, which had no effect on the composition of the sourdough microbial community. We tested three flour types, which had no effect on fungal composition, but did significantly shape the bacterial community, in line with similar studies (10, 24, 42, 43, 48). As the identity and relative abundance of strains and species in starters impart distinct functional traits to sourdough, such as aroma and acidification (49, 50), this suggests that flour type can shape a variety of sourdough traits.

It is unclear what mechanisms underlie the differences between fungal and bacterial responses to flour type. The flour types used here represent those commonly used flours in bakeries and homes and differ in their protein (~10–13%) and ash content (the amount of bran). Furthermore, the all-purpose and bread flours used in this experiment contained additives (vitamins and enzymes), and the all-purpose flour was bleached. These aspects of the flour can potentially influence the community composition and bread quality (51–55). The addition of enzymes, which break down the starch present in flour to simple sugar molecules for microbes to consume, likely evens out some of the differences between bread and all-purpose flours and whole wheat flour. However, we did not explicitly characterize amylase activity. Strains of the same yeast genus, *Kazachstania*, were identified in all flour types even as bacterial genera varied in composition. *Kazachstania* species, notably *K. humilis*, *K. unispora*, *K. exigua*, and *K. bulderi*, are some of the most commonly sampled yeast species in sourdough (12, 18, 21, 56, 57). Our results suggest that variation in flour contents did not alter which yeast was detected at the highest sequencing read abundance. However, we note that without culturing and counting, it is not possible to assess differences in viability between flour types or differences in the ratio of yeast to bacteria. In contrast to yeasts, the proportion of bacterial genera, as measured by sequence read abundance in each sourdough, was dependent on flour type, with *Companilactobacillus* present at higher abundance in whole wheat starters, *Lactiplantibacillus* in all-purpose starters, and *Levilactobacillus* more prevalent in bread flour starters. Again, without culturing these starters, it is challenging to interpret how relative read abundance translates into absolute abundance of these different bacterial taxa.

There are at least two non-exclusive explanations for the ubiquity of *Kazachstania* in our later-stage starters. First, the resources necessary for *Kazachstania* to outcompete other fungi may be sufficiently similar across flour types so as to allow it to dominate (in essence, a resource competition hypothesis). Second, *Kazachstania* may be more effective than other yeasts in the ecosystem associated with commercial flour production, such that it thrives as a function of its presence across flour types at relatively high propagule sizes. From an ecological perspective, this is a hypothesis about dispersal and propagule size, wherein the presence of *Kazachstania* in production facilities and flour serves to increase its dispersal into starters at a relatively large propagule size. In contrast to *Kazachstania*, the relative abundance (proportion of reads) and prevalence (number of starters) of *Saccharomyces* were low in our starters, at least throughout most of the study. This is reconcilable with the uneven distribution and low relative abundance of *Saccharomyces* in the flours we sampled, as well as previous observations that *Saccharomyces* rarely, if ever, establishes in sourdoughs from flour. In essence, *Saccharomyces* may be dispersal- or propagule-size–limited in young starters, but it may outcompete other yeasts through time, a possibility hinted at by the increase in *Saccharomyces* in the last days of our experiment. This competition may occur earlier in bakeries and other facilities, where the presence of *Saccharomyces* on bakers, in vessels, and on utensils increases the probability of its transfer. We note that we did not sample multiple batches of each flour, only replicated fermentations within the same batch of flour, so it is also possible that different batches of the same flour type could yield different prevalent taxa if dispersal limitation is important.

The differences in bacterial taxa among flour types have two possible explanations. The first relates to differences in colonization and hence dispersal and propagule supply. Some bacterial taxa in sourdough are clearly dispersal-limited. For example, *F. sanfranciscensis* is associated with older starters (11) and is likely passed from one starter to another. Conversely, the bacterial species *L. plantarum* and *L. brevis* are associated with newer starters, as one might expect of taxa that are not dispersal-limited, whether because they are conveyed in flour or because they are ubiquitous (11). In short, by reducing opportunities for dispersal of taxa via backslopping, we favored lineages that colonize readily. Even doing so, however, we saw differences in the taxa present in starters, at least as represented by relative abundance of sequencing reads. Several studies on acetic acid bacteria and lactic acid bacteria have not found strong associations between ecological niche and phylogenetic relationship (50, 58–60), so assessing whether there are unique adaptations to specific environments such as flour has been challenging. However, analysis of accessory gene content, genes that are variable in presence between different strains, suggests some level of strain adaptation to sourdough and other environments (50, 61–63). Exploration of accessory gene content in bacterial strains derived from starters of different flour types may illuminate how specific strains are able to compete for the resources present in a given flour starter.

The process of backslopping undoubtedly changes the microbial community in sourdoughs, as evidenced by the succession of organisms in the first weeks of sourdough formation in our own study and many others. It follows that feeding frequency could alter community dynamics by controlling the availability of nutrients and competition between species (25, 64, 65). While we did not detect any differences in microbial community based on feeding schedule, this may have been impacted by the similarity in frequency of our two feeding schedules (daily or every other day) and/or by the amount of starter that was maintained after discard. The ratio we used to passage starters during each feeding may select for microorganisms that survive in a highly acidic and potentially nutrient-limited environment. However, we note that the taxa we identified are all commonly found in sourdough. Furthermore, we refrigerated our sourdoughs over the weekend, which may have introduced further selection pressures. Refrigeration slows growth but is not expected to result in loss of viability. Refrigeration is commonly employed by home bakers, as are variable feeding frequencies and discard ratios, so the experimental design here captures relevant parameters of sourdough maintenance. Several previous studies suggest some modulation in microbial composition due to baking practices, including feeding frequency (12, 66). However, differences in parameters between our study and previous work make it challenging to draw conclusions. A larger-scale experiment that systematically tests the effect of feeding frequencies ranging from days to weeks—or similarly, a study that examines the inoculum-to-discard ratio—would be enlightening.

If we step back from our study to consider the bigger picture of sourdough starters, our experiment considers a narrowly circumscribed and relatively controlled case. Out of the tens of plant species employed in making starters, we considered just a single species: bread wheat (*Triticum aestivum*). While starters are made in many thousands of different ecological contexts (bakeries, outdoor settings in villages, tropical habitats, different biogeographic regions), we focused on a single context: a university lab. This controlled context presents advantages, disadvantages, and open questions. The disadvantage is that it limits the potential for colonization in ways that are not reflective of many or most contexts in which sourdough starters are actually produced (13, 14). We limited exposure to the hands of bakers, house microbiota, and previously colonized vessels. Yet, even once we limit these additional influences, we still see variation, both among replicates and with regard to treatments. Is this variation purely stochastic, or are there hidden differences among replicates that we have missed? As for the advantages, our experiment allowed a focus on the differences due to narrow permutations. While feeding frequency had no effect, given the details of our experiments, flour type did, at least on bacteria. Laboratory sourdough microcosms thus offer a complement to the nature of kitchens and bakeries, one in which we can see influences that are less visible in nature, such as those we have documented here.

## MATERIALS AND METHODS

### Sourdough starter setup and passaging

We selected three flours (all Gold Medal brand, distributed by General Mills): whole wheat (ingredients: whole wheat flour); enriched bleached all-purpose (ingredients: bleached wheat flour, niacin, iron, thiamine mononitrate, riboflavin, enzymes, folic acid); and enriched unbleached bread flour (ingredients: wheat flour, niacin, iron, thiamin mononitrate, riboflavin, enzymes, folic acid). Sourdough starters were initiated by combining 100 g of flour and 100 g of DI water and mixing with a spatula in sterilized 250 mL mason jars with lids. Starters were fed either daily or every other day, and all starters were stored in a 4°C refrigerator over the weekend. Each flour type and feeding schedule was done in triplicate, for a total of 18 independent starters. The starters were fed either daily (every ~24 h) or every other day (every ~48 h) by discarding 100 g of starter and replacing it with 50 g of the appropriate flour type and 50 g of DI water, which was mixed with a spatula. Samples were taken for DNA extraction and glycerol stock archives once per week prior to feeding. Approximately 1 mL of dough from each starter was extracted and saved in a 1.5 mL centrifuge tube at −80°C for later DNA extraction. Glycerol stocks were made at the same time by extracting 1 mL of dough and vortexing it in 1 mL of 30% glycerol; glycerol stocks were stored at −80°C. Five time points were collected from each starter starting on day 2 after the starter was initiated and ending after 28 days of passaging.

### Amplicon DNA sequencing and analysis

DNA was extracted using the QIAGEN DNeasy PowerSoil Pro Kit and prepared for amplicon sequencing following the Illumina 16S/ITS guide (67). Primers used for library creation can be found in Table S1 (as described in reference 31). The 16S and ITS libraries were pooled and run on an Illumina MiSeq 250 bp PE (Duke University Sequencing Core). The number of reads for each sample is provided in Table S2.

All downstream analysis was performed in the R environment (68). We used Cutadapt to remove the primers (69). We then used the DADA2 pipeline v1.16 (70) to resolve the exact amplicon sequence variants (ASVs). For the ITS data, we used maxEE = 2 for both forward and reverse reads, truncQ = 2, and minLen = 50. For the 16S data, reverse reads were discarded due to poor quality. We used maxEE = 2, truncQ = 2, and truncLen = 160. Next, we merged paired-end reads and removed chimeras and any contamination. Genus-level taxonomy was assigned using the Silva database version 138.1 for 16S reads (71) and with the UNITE database version 9 for ITS reads (72) (Tables S3 and S4). We filtered out 16S reads assigned to chloroplast or mitochondria. We performed rarefaction analysis of all data sets for both 16S and ITS data sets with the vegan R package (73) and determined that the minimum library size was sufficient to reach the asymptotes for richness for alpha diversity analysis.

We used the vegan R package to calculate alpha diversity, measured as observed (ASVs) richness and Shannon index, for each treatment mentioned above (73), based on ASVs rarefied to the minimum library size. For beta diversity and individual taxa associations, ASV read counts were normalized to relative abundances (proportions of total reads), instead of rarefying. For statistical analysis, we used two-way ANOVA to assess the effects of feeding schedule (daily, every other day) and flour type (all-purpose, bread flour, whole wheat flour) as two factors on fungal and bacterial composition and alpha diversity. Then, Tukey’s HSD test was employed as a post-hoc test to determine which specific groups differed from each other. The Kruskal–Wallis H test, with a Bonferroni correction, was conducted to determine if the effect of elapsed day (0, 7, 14, 21, 28) was significant on sourdough microbial and fungal communities (74). We used the vegan R package to calculate beta diversity among the three flour types using Bray–Curtis dissimilarity (73). We conducted permutational multivariate analysis of variance (PERMANOVA) using the adonis2 function in the vegan R package to test the effects of flour type and feeding frequency on the composition of bacterial and fungal communities (73).

## Data Availability

The 16S and ITS sequences are available at NCBI SRA under PRJNA1292877. Taxa tables are available in the supplement (Tables S3 and S4).

## SUPPLEMENTAL MATERIAL

Supplemental figures spectrum.02380-25-s0001.docx Figures S1 to S6.

Supplemental tables spectrum.02380-25-s0002.xlsx Tables S1 to S7.

ASM does not own the copyrights to Supplemental Material that may be linked to, or accessed through, an article. The authors have granted ASM a non-exclusive, world-wide license to publish the Supplemental Material files. Please contact the corresponding author directly for reuse.
