# Supplementary material for: The role of flour type and feeding schedule on the sourdough microbiome
Source: Microbiol Spectr. 2025 Nov 25;14(1):e02380-25. doi: 10.1128/spectrum.02380-25 (PMC12772405; doi:10.1128/spectrum.02380-25)
Supplement: Supplemental figures — Figures S1 to S6. [file spectrum.02380-25-s0001.docx]

**Figure S1: Alpha diversity of microbial communities in different flour types prior to creation of sourdough starter**

**A. Fungal B. Bacterial**


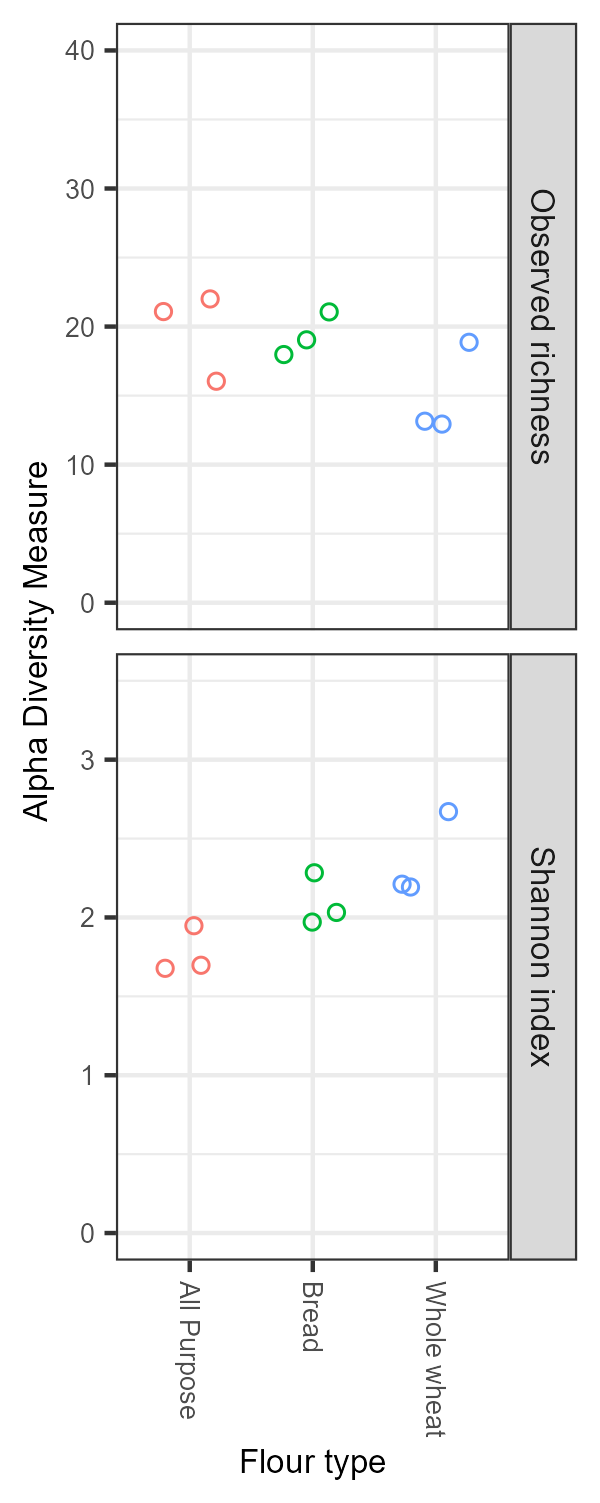

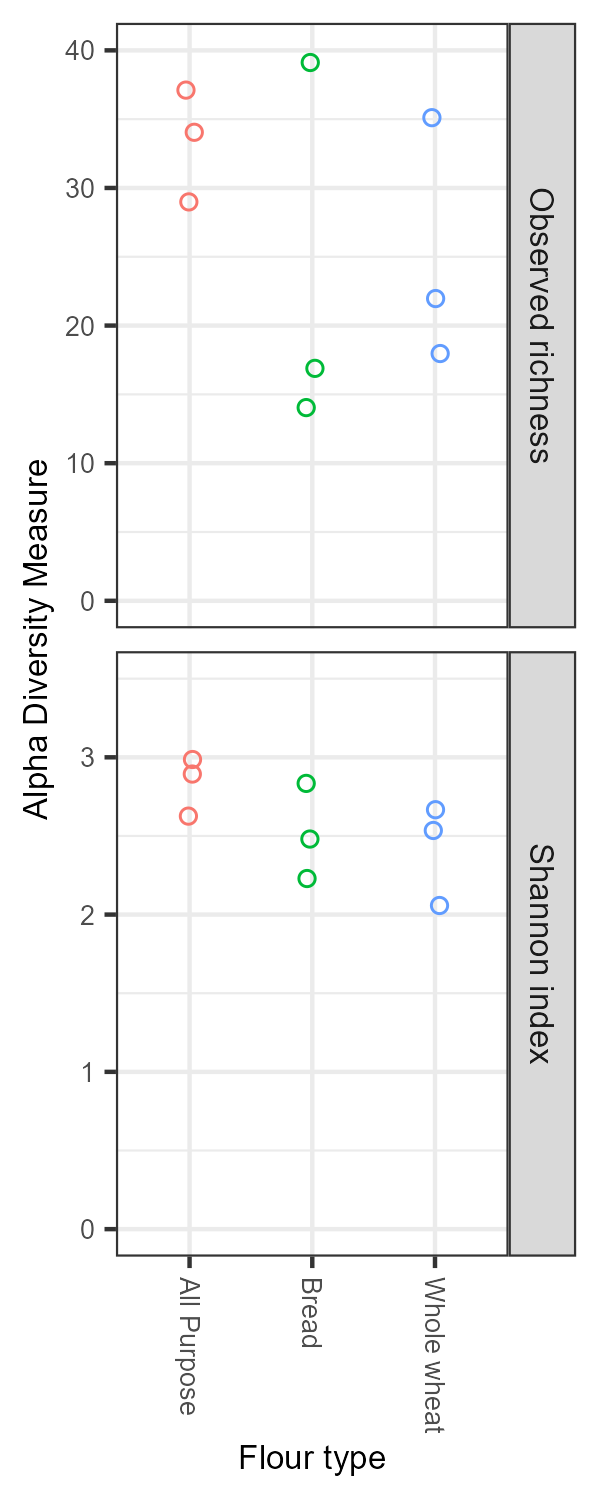


(A) Fungal Observed Richness (Kruskal-Wallis, chi-squared = 3.0313, df = 2, p =0.2197) and Shannon Index (Kruskal-Wallis, chi-squared = 5.9556, df = 2, p = 0.05091) measures of alpha diversity in All Purpose, Bread, and Whole wheat flour prior to the creation of sourdough starters. (B) Bacterial Observed richness (Kruskal-Wallis, chi-squared = 1.1556, df = 2, p =0.5611) and Shannon Index (Kruskal-Wallis, chi-squared = 3.2889, df = 2, p = 0.1931) measures of alpha diversity in All Purpose, Bread, and Whole wheat flour prior to the creation of sourdough starters.

**Figure S2: Beta diversity of microbial communities in flour types prior to creation of sourdough starter**

**A. Fungal**


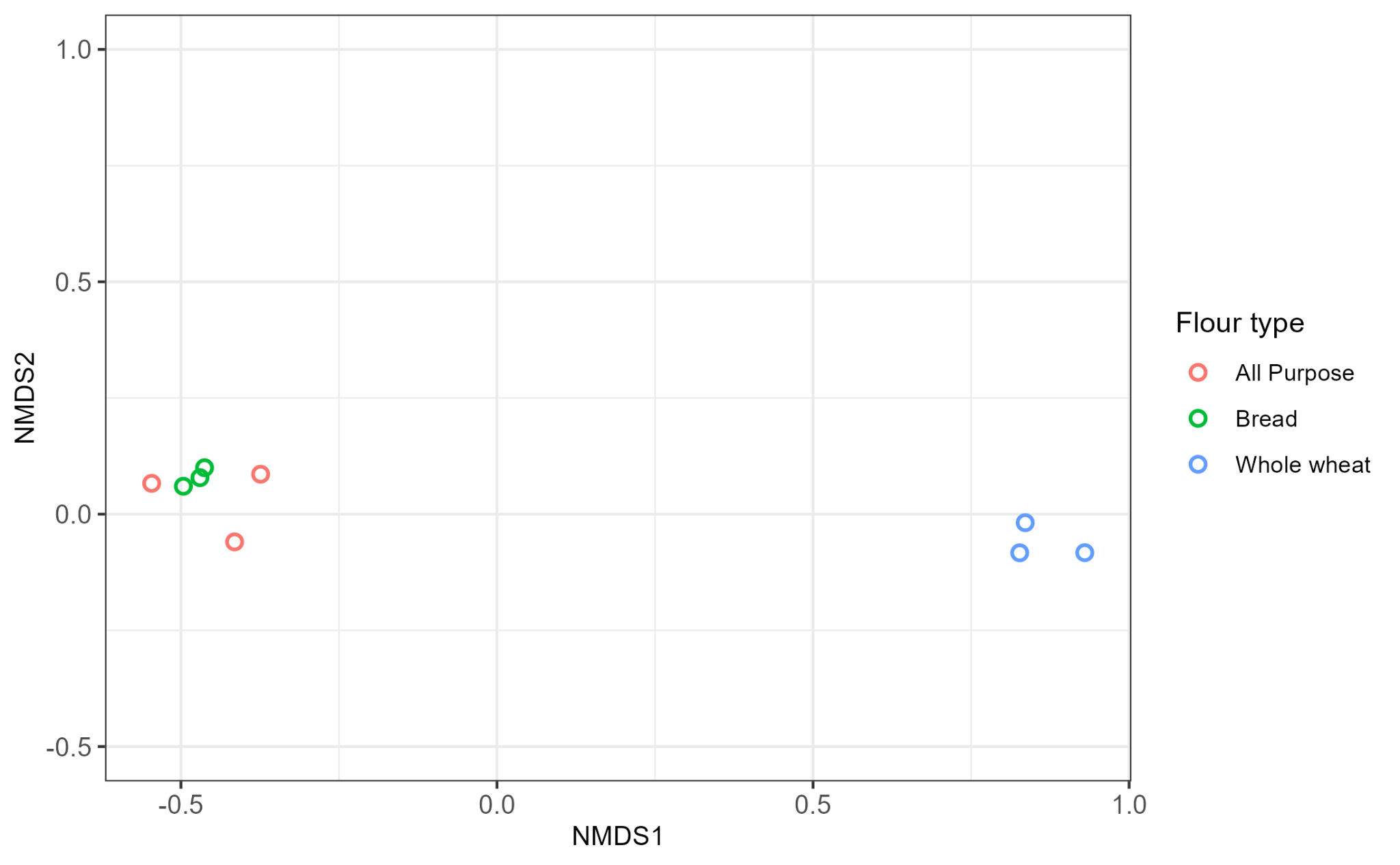


**B. Bacterial**


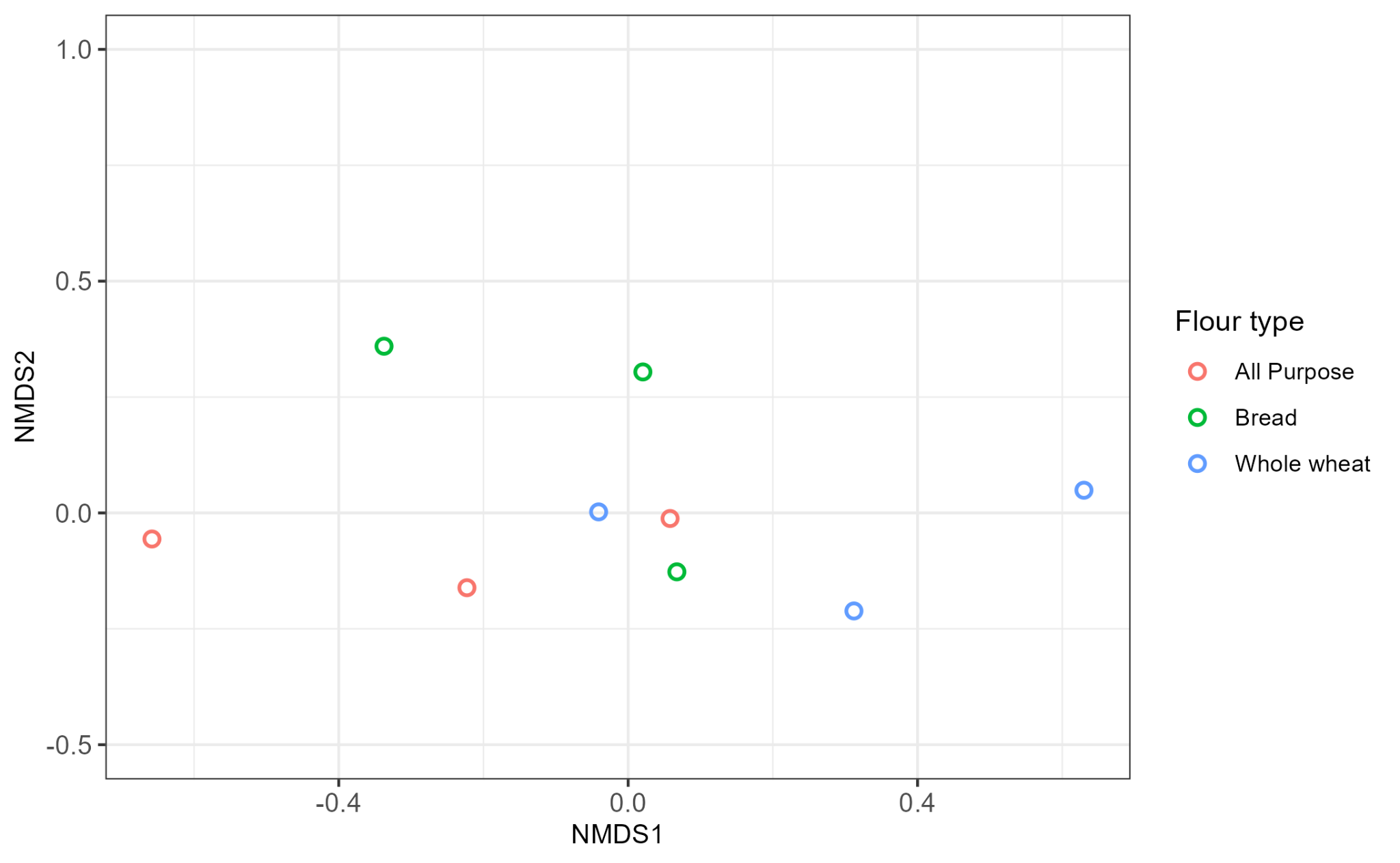


Bray-Curtis Non-metric multidimensional scaling (NMDS) plot of (A) fungal (p= 0.002997 by PERMANOVA) and (B) bacterial (p=0.2338 by PERMANOVA) communities in different flour types prior to the creation of sourdough starters.

**Figure S3: OR heat map of fungal and bacterial abundance over 28 days**

**A. Fungal**  **B. Bacterial**


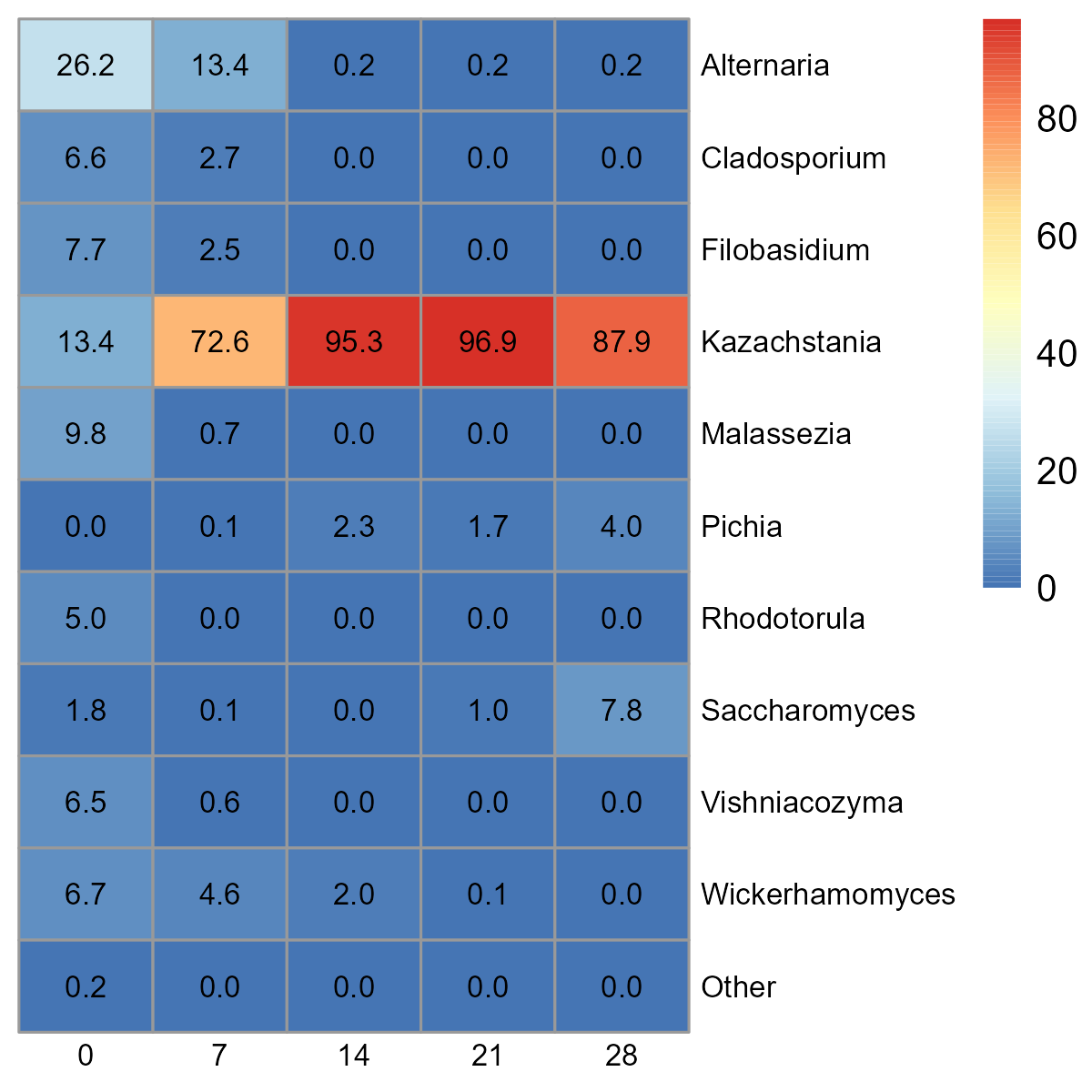

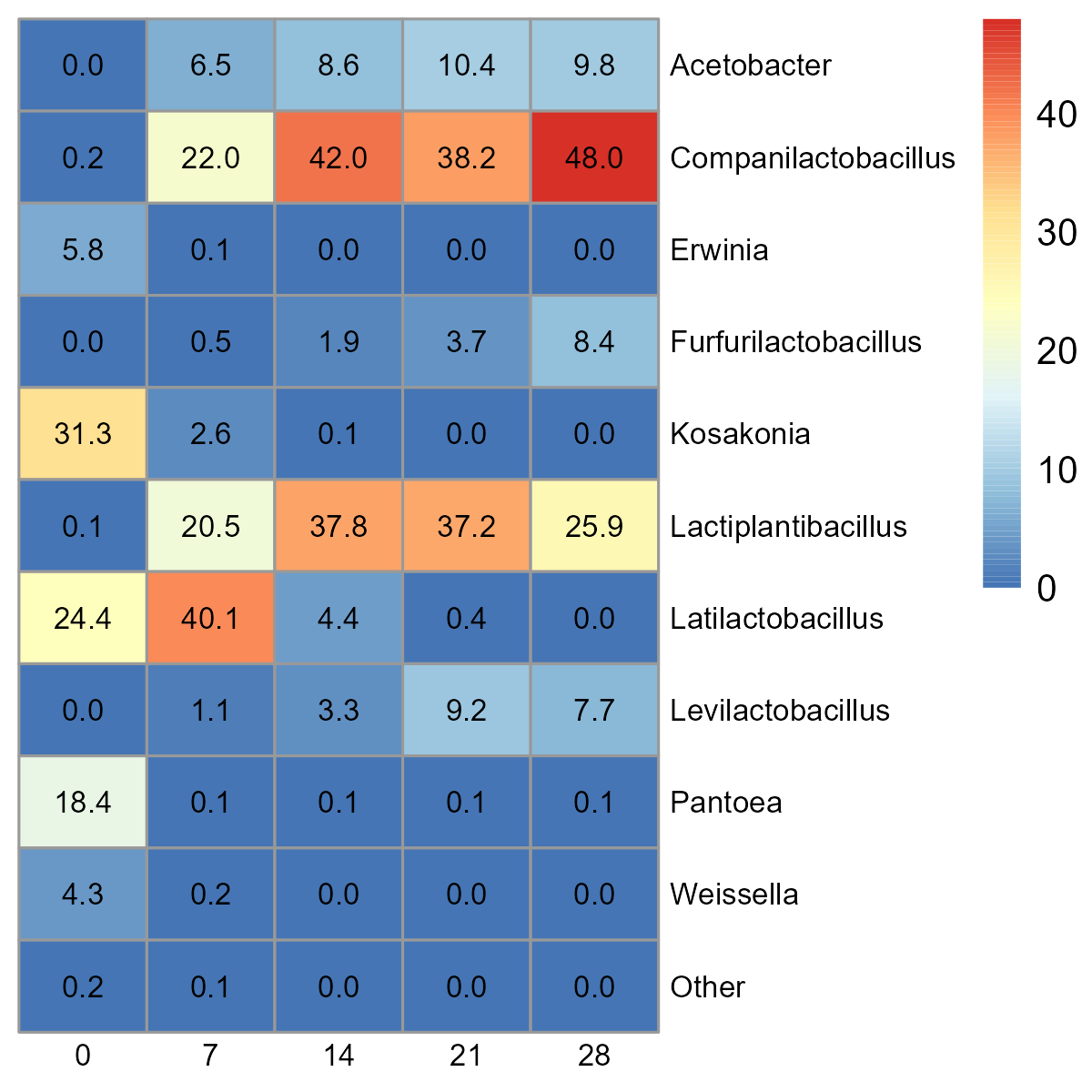


Patterns of fungal (A) and bacterial (B) succession of top 10 genera across all sourdough starters. The numerical values in each cell represent the relative abundance (%) of each genus on respective days (0, 7, 14, 21, 28).

**Figure S4: Alpha diversity measures of fungal and bacterial composition after 28 days**

**A. Fungal B. Bacterial**


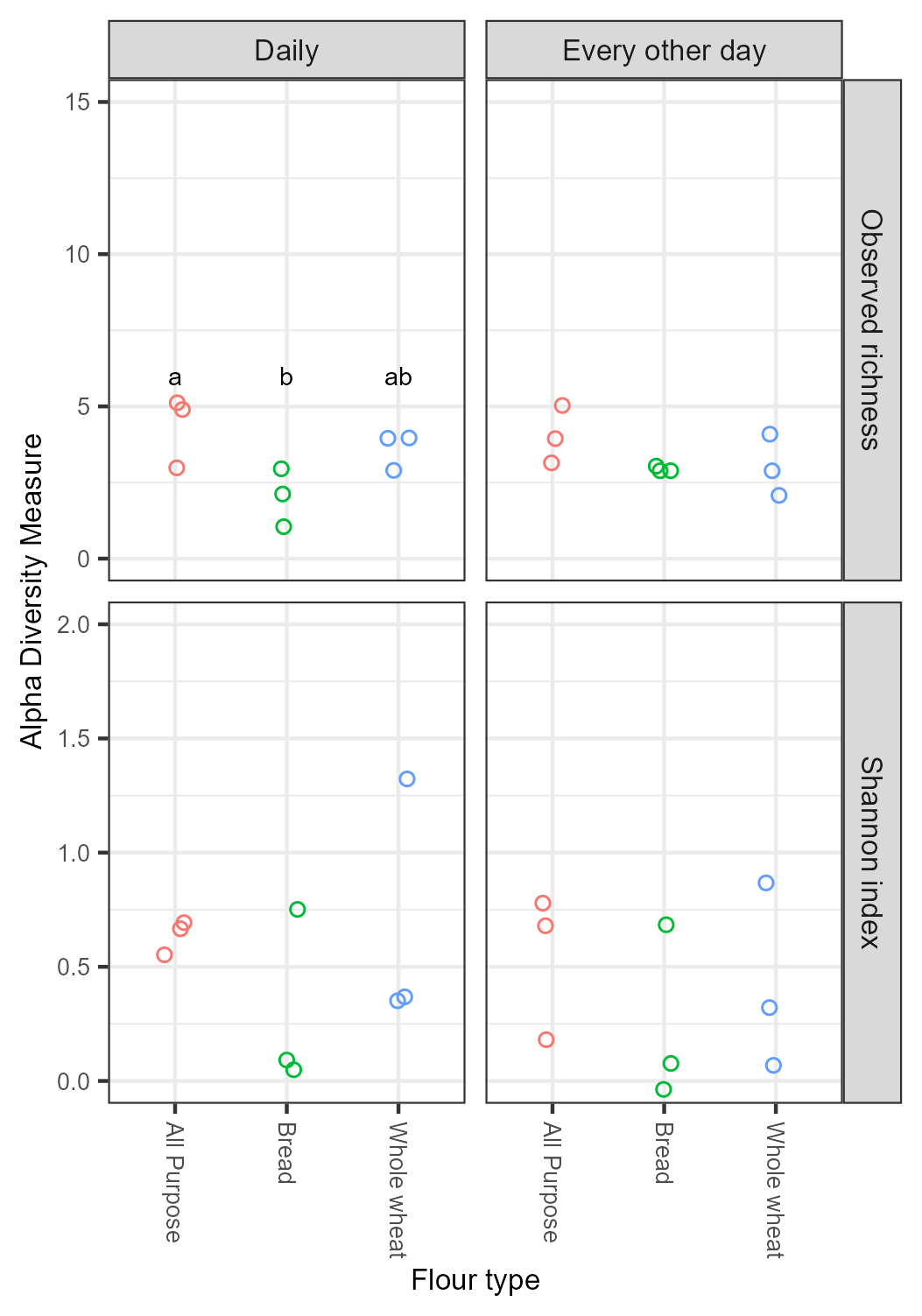

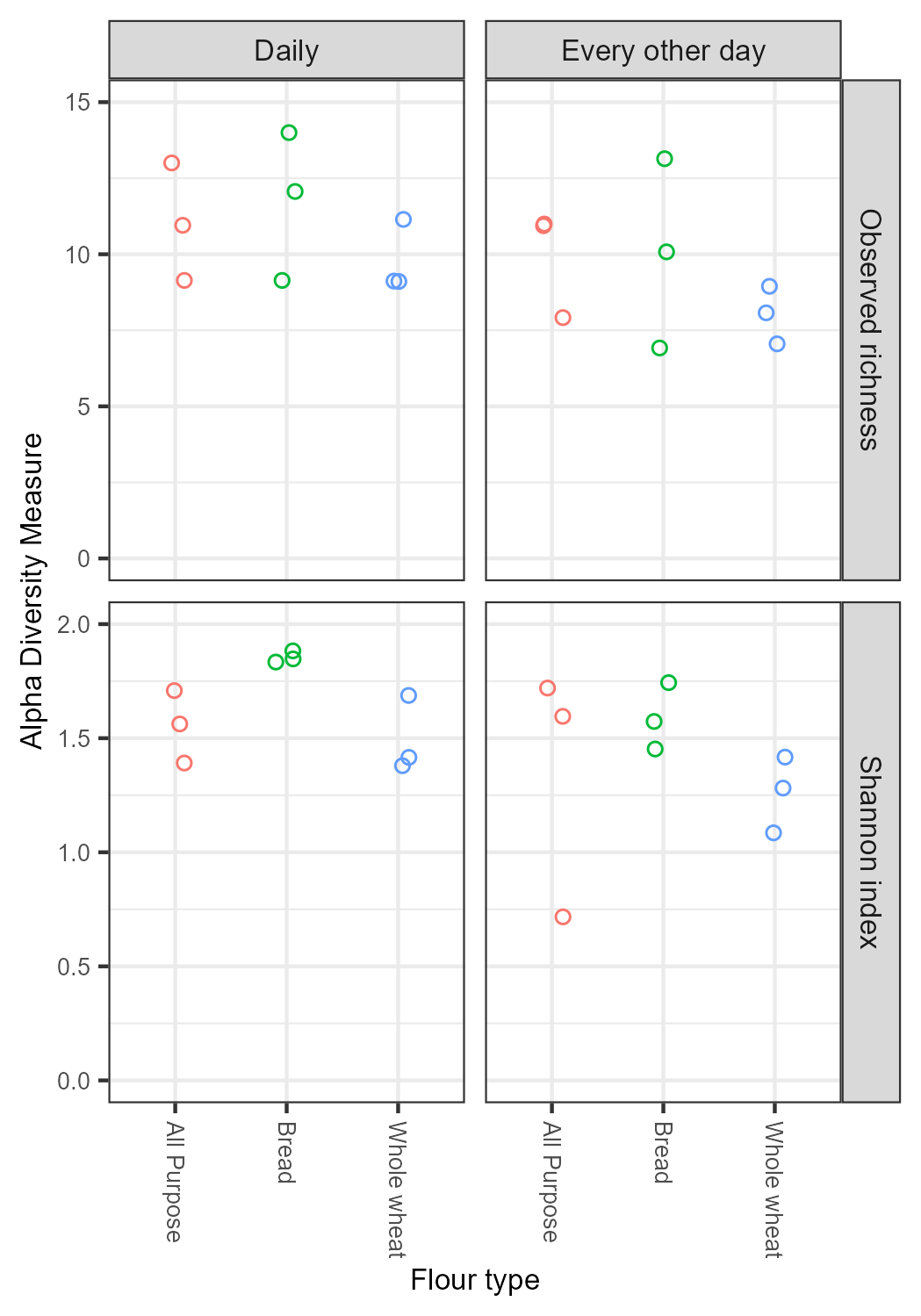


Fungal (A) and Bacterial (B) Observed Richness and Shannon Index measures of alpha diversity on day 28 in samples with different feeding frequencies and different flour types. Fungal analyses (Two-way ANOVA, followed by post-hoc Tukey HSD at 95% family-wise confidence level) revealed a significant difference in Observed Richness when comparing daily-fed All Purpose flour and Bread flour sourdough (p-adj = 0.01339). No other comparisons within fungal or bacterial datasets yielded significant results. P-values for all analyses are reported in Table S6.

**Figure S5: Beta diversity of fungal and bacterial composition after 28 days**

1. **Fungal**

**
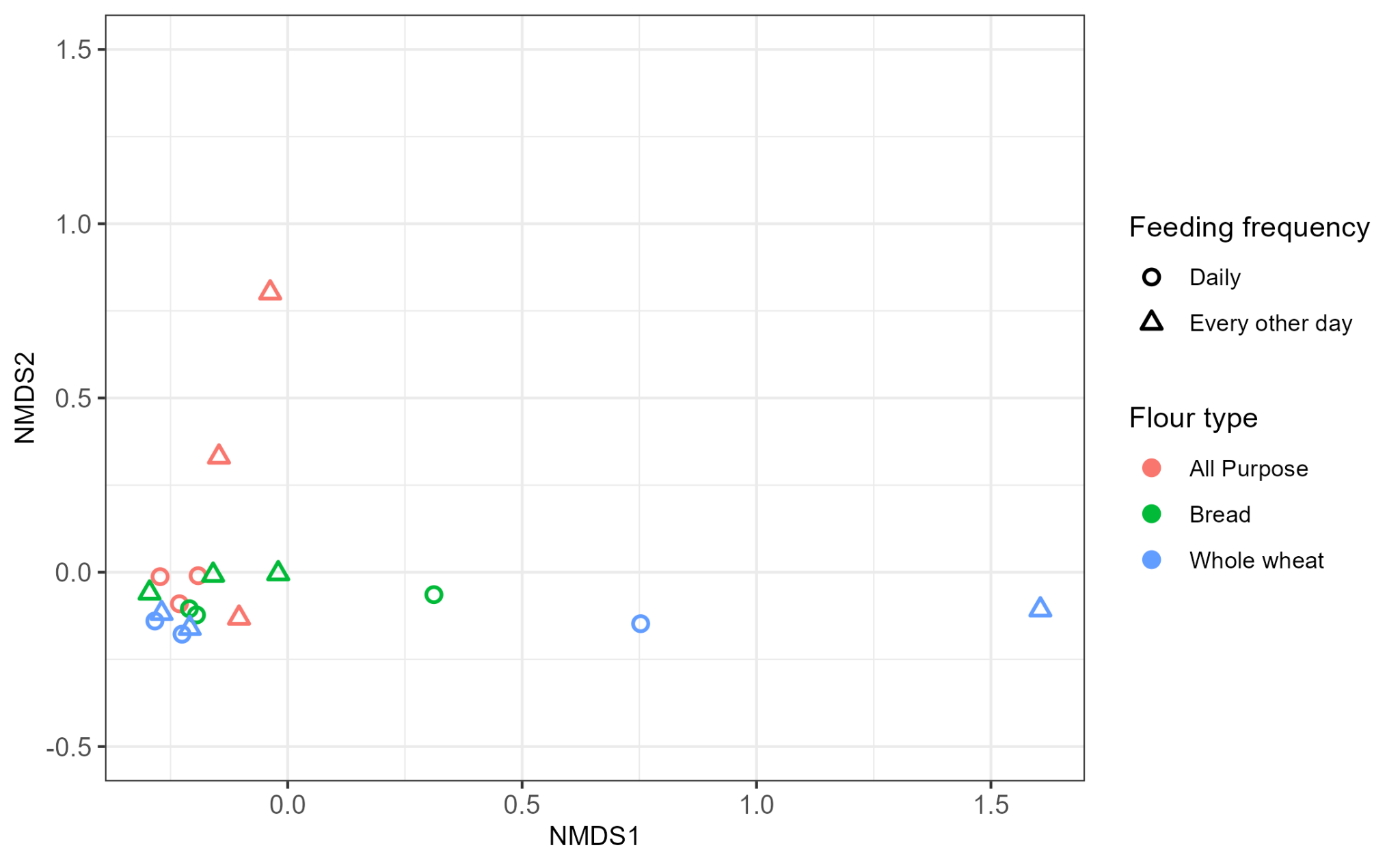
**

1. **Bacterial**

**
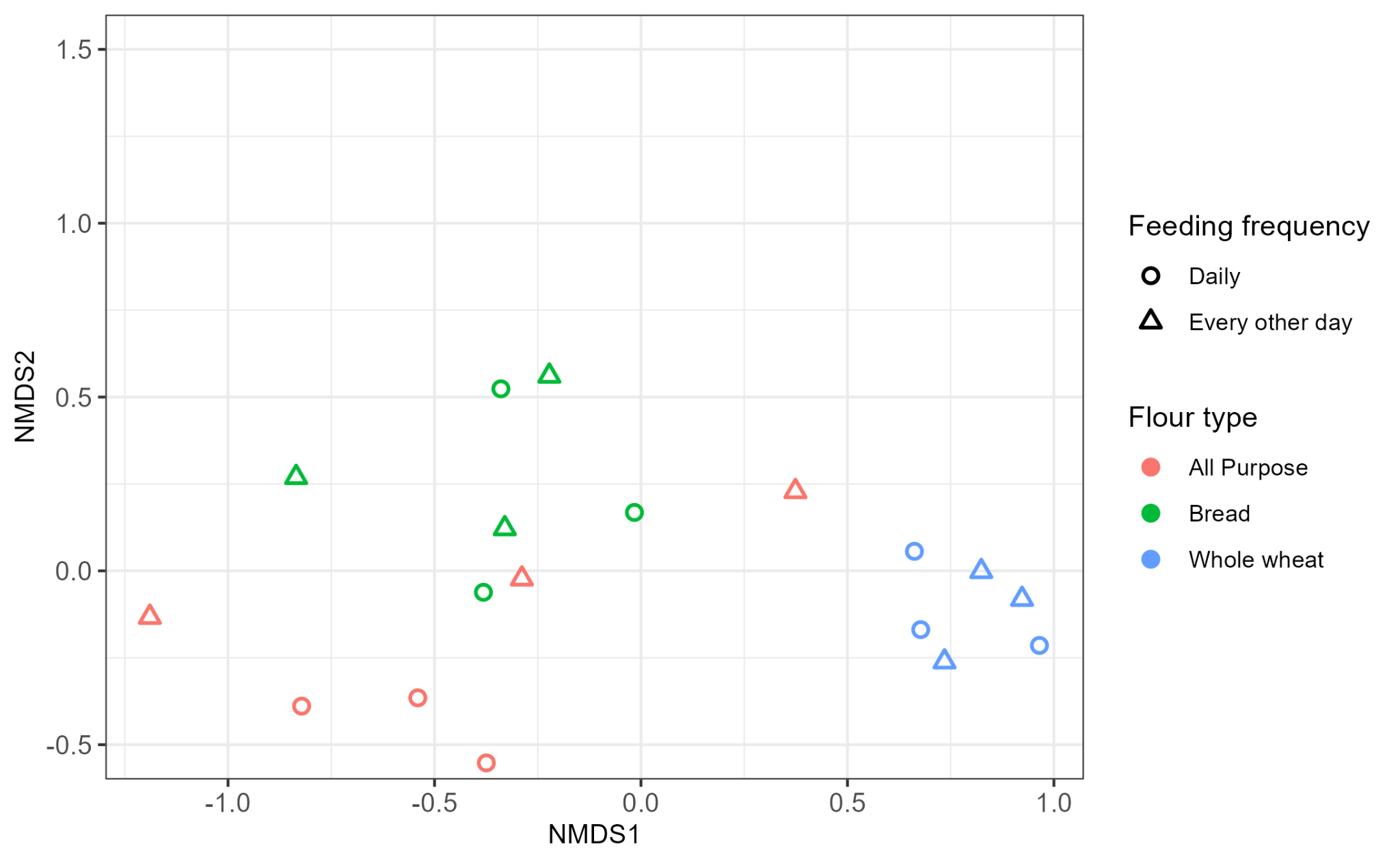
**

Non-metric multidimensional scaling (NMDS) plot of pairwise Bray-Curtis dissimilarities for Fungal (A) and bacterial (B) communities in samples with different feeding frequencies and different flour types on day 28 of feeding. Fungal analyses yielded flour (p=0.06194), feeding frequency (p=0.56743) and flour*feeding_frequency (p=0.68731) by PERMANOVA. Bacterial analyses yielded flour (p=0.000999), feeding frequency (p=0.111888) and flour*feeding_frequency (p=0.374625) by PERMANOVA.

**Figure S6: Alpha diversity measures over time**

**A. B.**
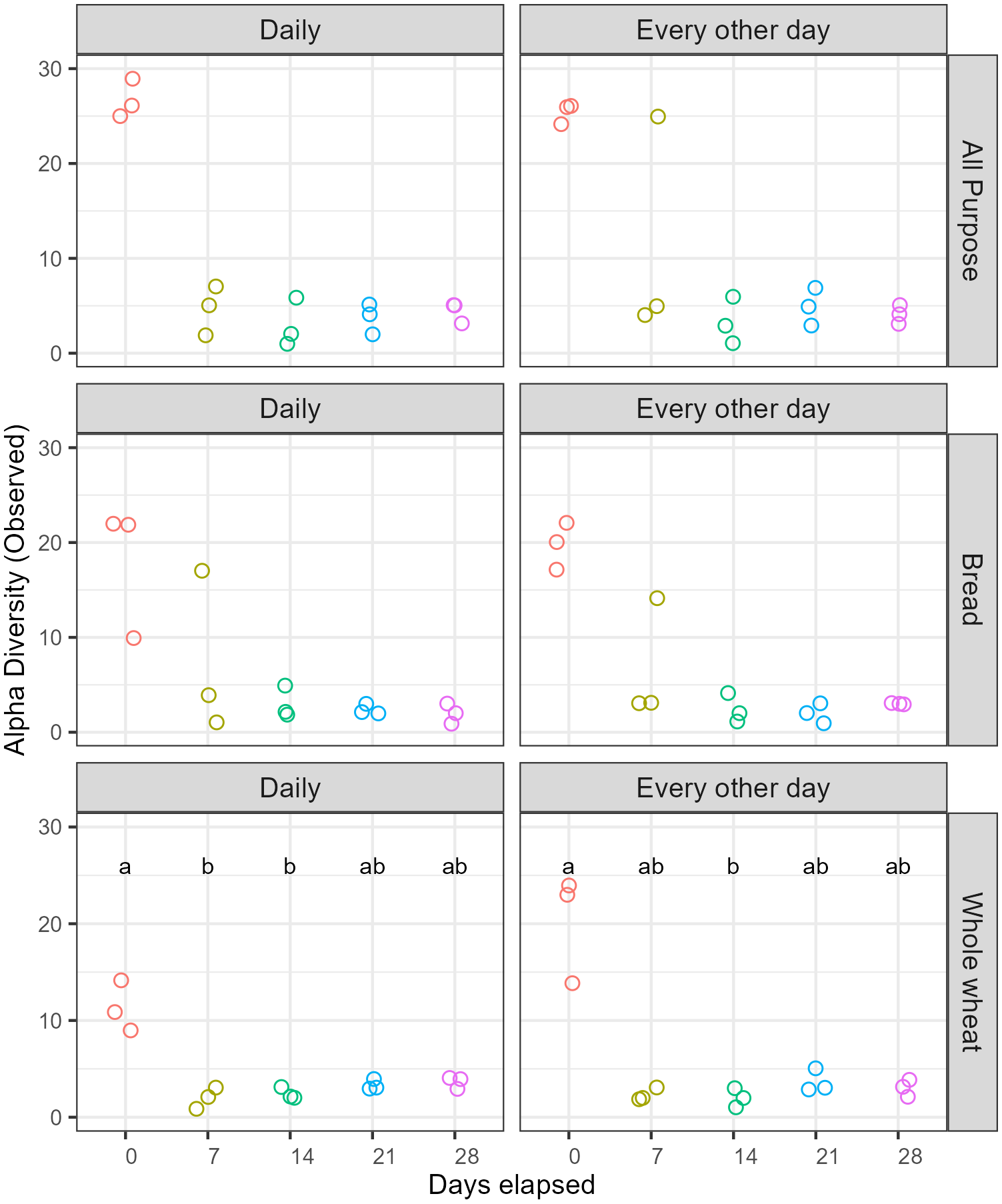

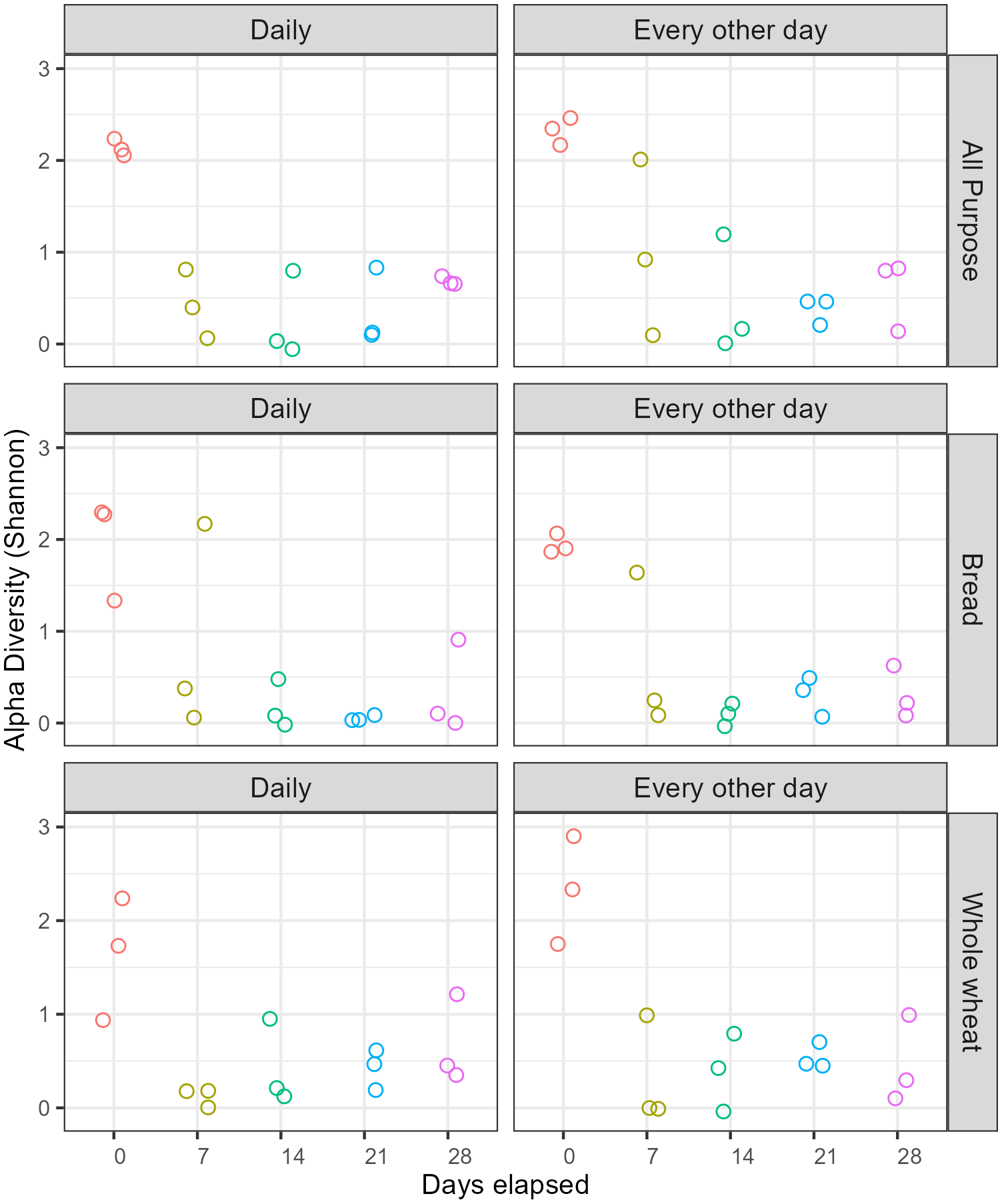


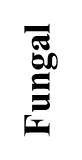

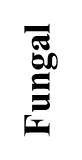


**C. D.**
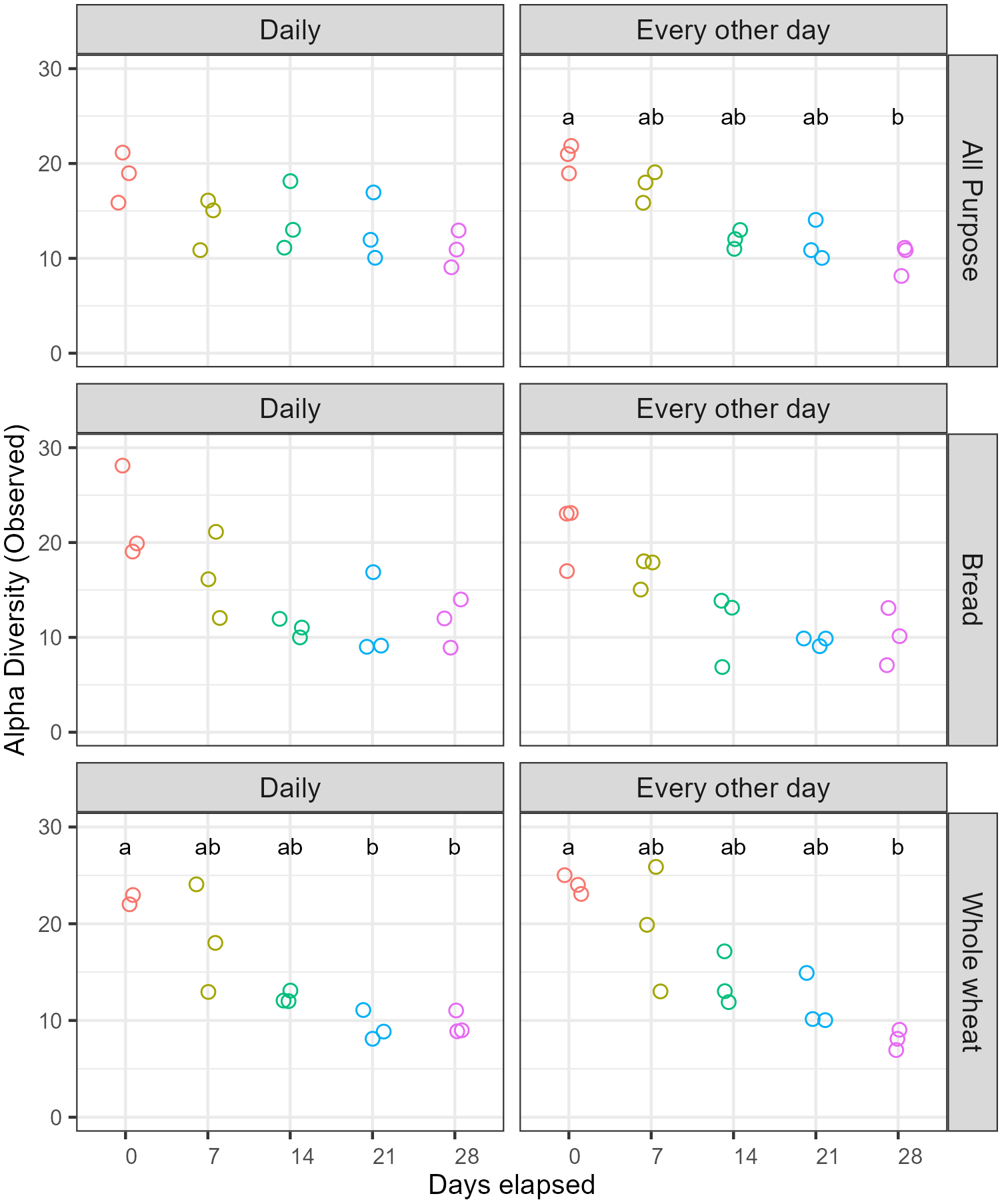

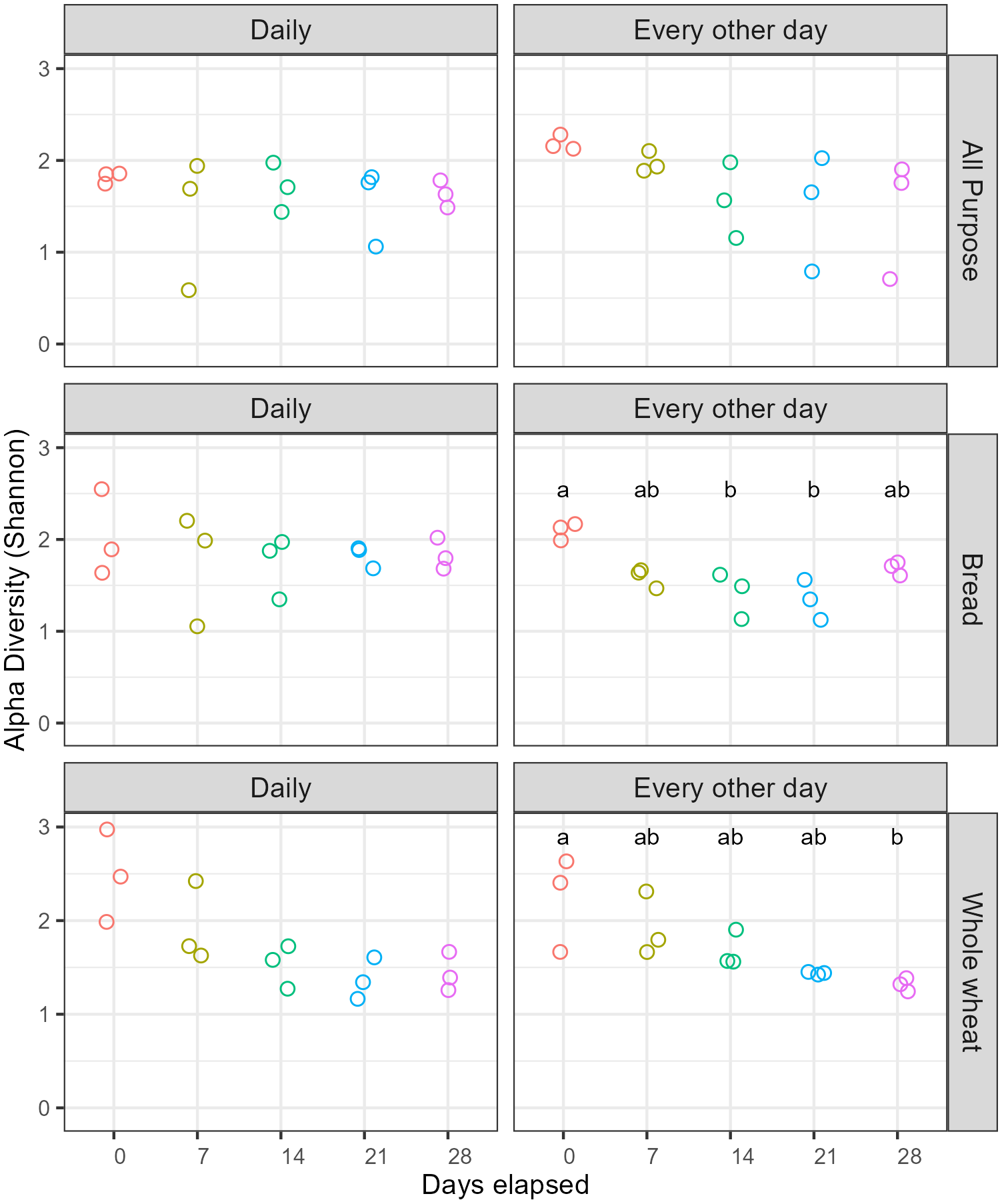


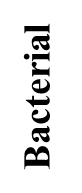

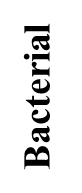


Fungal (A,B) and Bacterial (C,D) Observed Richness and Shannon Index measures of alpha diversity over time in All Purpose, Bread, and Whole wheat sourdough starters with feeding schedules of either Daily or Every other day. Significant differences were detected via Kruskal-Wallis test, followed by a post-hoc Dunn’s test with a Bonferroni correction. P-values for all analyses are reported in Table S7.
